# Adaptation of a social vulnerability index for measuring social frailty among East African women

**DOI:** 10.1186/s12889-022-12597-z

**Published:** 2022-01-24

**Authors:** Sandeep Prabhu, George Wanje, Brenda Oyaro, Francis Otieno, Kishor Mandaliya, Walter Jaoko, R. Scott McClelland, Wayne McCormick, Melissa K. Andrew, Frances M. Aunon, Jane M. Simoni, Susan M. Graham

**Affiliations:** 1 University of Washington, Seattle, WA USA; 2 University of Nairobi, Nairobi, Kenya; 3 Coast General Teaching and Referral Hospital, Mombasa, Kenya; 4 PathCare Laboratory, Mombasa, Kenya; 5 Dalhousie University, Halifax, Canada; 6 Yale School of Medicine, New Haven, CT USA; 7 Veterans Affairs Connecticut Healthcare System, West Haven, CT USA

**Keywords:** Older women, HIV/AIDS, Social frailty, Kenya, Mombasa

## Abstract

**Background:**

The number of older women living with HIV in Africa is growing, and their health outcomes may be adversely impacted by social frailty, which reflects deficits in social resources that accumulate over the lifespan. Our objective was to adapt a Social Vulnerability Index (SVI) originally developed in Canada for use in a study of older women living with or without HIV infection in Mombasa, Kenya.

**Methods:**

We adapted the SVI using a five-step process: formative qualitative work, translation into Kiswahili, a Delphi procedure, exploration of potential SVI items in qualitative work, and a rating and ranking exercise. Four focus group discussions (FGD) were conducted (three with women living with HIV and one with HIV-negative women), and two expert panels were constituted for this process.

**Results:**

Themes that emerged in the qualitative work were physical impairment with aging, decreased family support, a turn to religion and social groups, lack of a financial safety net, mixed support from healthcare providers, and stigma as an added burden for women living with HIV. Based on the formative FGD, the expert panel expanded the original 19-item SVI to include 34 items. The exploratory FGD and rating and ranking exercise led to a final 16-item Kenyan version of the SVI (SVI-Kenya) with six domains: physical safety, support from family, group participation, instrumental support, emotional support, and financial security.

**Conclusions:**

The SVI-Kenya is a holistic index to measure social frailty among older women in Kenya, incorporating questions in multiple domains. Further research is needed to validate this adapted instrument.

## Introduction

In sub-Saharan Africa, approximately 1 in 8 persons living with HIV infection (PLWH), including 1 in 10 adults receiving antiretroviral therapy (ART), are aged 50 or older [1, 2]. This older population is fast growing, with HIV prevalence among those aged 50 and older expected to double by 2040 [1, 2]. Because women in Africa are disproportionately affected by the HIV epidemic, representing 59% of PLWH, this older population of PLWH is likely to be majority female [3]. While studies have shown that older African women report more chronic health conditions and have higher physical frailty rates than men, less is known about the health of older women living with HIV relative to their peers without HIV [4, 5].

In Kenya, a country where over a third of the population still lives in poverty, HIV care is free but care for other medical conditions is harder to access; consequently, families and other social contacts are critical sources of support for women’s overall health and psychosocial needs [6–9]. In this respect, social frailty, defined as “continuum of being at risk for losing, or having lost, resources that are important for fulfilling one or more basic social needs during the life span,” may be an important concept in these settings [10]. Social frailty may be a key area in which older women living with HIV may differ from their peers, given pervasive HIV stigma in the community and higher HIV prevalence among widows [11].

The Social Vulnerability Index (SVI) developed by Andrew and colleagues has been used to investigate associations between social frailty and health outcomes including cognitive decline and mortality [12, 13]. Very little work has explored whether social frailty, as captured by the SVI, provides important information about women’s ability to meet personal care goals and optimize their health outcomes as they age. Optimal health outcomes may depend on the social resources available to women, not only in terms of instrumental support (i.e., tangible help such as shopping or preparing meals), but also in terms of emotional support and motivation for care engagement [14]. Deficits in social resources could lead to adverse outcomes, whether related to HIV care or the management of other chronic health conditions.

In preparation for a study of social frailty among HIV-positive and HIV-negative women aged 40 and older living in Mombasa, Kenya, we aimed to investigate the relevance of the SVI items in this context and identify any new items women and their providers would consider more relevant. While Bunt et al. adapted the SVI for use in the Dutch context, we felt a modified process was needed in Kenya to take literacy and the cultural context into account, leading to a unique adapted instrument [15]. In this manuscript, we describe our process for evaluating SVI items and adapting this inventory to the Kenyan context.

## Methods

### Study overview

We based our approach initially on a stepwise process used by Bunt and colleagues for adaptation of the SVI for use in the Netherlands [15]. In addition, we conducted focus group discussions (FGD) with older women to inform the initial research focus and gain insight into the lived experiences of the target population, including their views on what made women socially vulnerable as they aged. The stepwise process proceeded as described below.

### Step one: formative qualitative work

Based on the information, motivation, behavioral skills (IMB) adherence model of Fisher and colleagues [16], we developed a semi-structured interview guide assessing HIV knowledge, motivation to take ART, and skills and cues for care engagement and pill taking, while exploring the role of social support from family, friends, community organizations, and healthcare providers. This topic guide was used to conduct two FGDs in November 2017 with women aged 40 and over living with HIV in urban and peri-urban Mombasa. These two FGDs were recorded, transcribed, and translated, and their findings informed our decision to focus on social vulnerability as a key area of research on older women’s health.

### Step two: translation of the SVI into Kiswahili

Two experienced Kenyan professional translators fluent in English and Kiswahili separately translated the 19-item SVI as used by Armstrong and colleagues [13] into Kiswahili. Next, the two translations were synthesized by a research team member fluent in Kiswahili (GW), who discussed differences with the two translators to obtain consensus. The translated SVI was then back translated, and the new English version compared to the original SVI for discrepancies, updating the Kiswahili version as needed.

### Step three: Delphi procedure

We formed a group of 5 experts (local providers or community representatives) fluent in both English and Kiswahili who were selected based on their expertise on the health and social situation of older Kenyan women. This group was convened in October 2019 for a detailed review the 19-item SVI using a Delphi procedure. The experts were asked to consider each SVI item in the context of their own lived experience and in relation to findings from the formative FGD results, which were presented in summary form. Ideas for items not currently included in the SVI that were important in the Kenyan context (e.g., food security, literacy) were suggested and discussed. Items which were agreed to be potentially important were added to a working document with all items being considered for the adapted version, referred to as the “SVI-Kenya.”

### Step four: exploration of potential SVI-Kenya items in qualitative work

Based on the input from our expert committee, we developed a new topic guide to examine the concept of social frailty in further depth in two additional FGDs among women aged 40 and over, one with women living with HIV and the other with HIV-negative women. This topic guide broke the different potential SVI-Kenya items into several domains to enable discussion of similar concepts, including living situation, family, other relationships, general support, socializing, and physical impairment. These FGDs were conducted in late 2019 and early 2020, and were recorded, transcribed, and reviewed by the study team prior to reconvening the expert panel.

### Step five: rating and ranking of potential SVI-Kenya items

The final step of our adaptation process was to cull the expanded list of SVI-Kenya items. Our panel was joined by a female Kenyan clinician with > 5 years of experience providing care to women of a variety of ages (BO) and the Kenyan sociobehavioral scientist (GW) who conducted the FGD. After presentation of the preliminary results of the two FGD conducted in step four to explore women’s views on social frailty, these 7 individuals were asked to rate the importance of each of the candidate SVI-Kenya items on a scale of 1–5, with importance rated as 1 being “very important,” 2 “important,” 3 “neutral,” 4 “unimportant,” and 5 being “should be removed. In addition, panel members were asked to rank the items within each domain from most to least important, assigning 1 to the highest ranked item, 2 to the next most important, etc. Only items with an average importance rating less than 2 were considered for retention. For parsimony, averaged rankings were then used to select only the most highly ranked items within a given domain by group consensus.

### Thematic analysis of qualitative work

All four recorded FGDs were transcribed verbatim and translated into English. An iterative process was employed to identify themes related to social frailty and health. Transcripts were first read several times by the three investigators (SMG, SP, and GW) to understand the content and reflect on the research objective. A draft codebook was developed and refined throughout the inductive coding process. The three investigators applied the first draft of the codebook separately to each transcript. They discussed areas where the codebook needed refinement, until consensus was reached, and the codebook updated. A second round of coding was then done on all transcripts by the three investigators, using the final codebook. Atlas.ti (a qualitative data analysis software package) and manual coding were both used on to analyze the transcripts. Content analysis was employed to organize emergent themes and sub-themes.

## Results

### Study population

Table 1 presents demographic characteristics of participants in the four FGDs: two with HIV-positive women in 2017 (FGD 1 and 2), one with HIV-negative women in 2019 (FGD 3), and one with HIV-positive women in January 2020 (FGD 4). The median age of the 34 FGD participants was 52 (range, 42 to 66). The median number of years of education was 8, with half of the participants not progressing beyond primary education. Table 2 contains demographics of the expert panel participants, who were all Kenyan, aged between 28 and 64, and had completed secondary education plus additional training in their field of expertise. The two expert panels included 4 nurses, 2 health facility receptionists, 1 counsellor, 1 laboratory technician, 1 clinician, and 1 sociobehavioral scientist.

**Table 1:** Characteristics of FGD participants

Characteristic N (%) or median (range)	FGD 1 (11/24/17) *n*=7	FGD 2 (11/27/17) *n*=8	FGD 3 (11/29/19) *n*=11	FGD 4 (1/21/20) *n*=8	Overall *n*=34
HIV status	Positive	Positive	Negative	Positive	--
Age in years	53 (50–55)	52 (50–62)	51 (42–66)	53 (50 –56)	52 (42–66)
Country of birth					
*Kenya*	5 (71.4)	7 (87.5)	11 (100)	7 (87.5)	30 (88.2)
*Uganda*	2 (28.6)	0 (0)	0 (0)	0 (0)	2 (5.9)
*Tanzania*	0 (0)	1 (12.5)	0 (0)	1 (12.5)	2 (5.9)
Religion					
*Protestant*	4 (57.1)	4 (50)	5 (45.4)	5 (62.5)	18 (52.9)
*Catholic*	2 (28.5)	3 (37.5)	2 (18.2)	2 (25)	9 (26.5)
*Moslem*	1 (14.2)	1 (12.5)	4 (36.4)	1 (12.5)	7 (20.6)
Marital status					
*Never married*	0 (0)	1 (12.5)	3 (27.3)	0 (0)	4 (11.8)
*Currently married*	0 (0)	1 (12.5)	4 (36.4)	4 (50)	9 (26.5)
*Widowed*	7 (100)	6 (75)	2 (18.2)	4 (50)	19 (55.9)
*Divorced*	0 (0)	0 (0)	2 (18.2)	0 (0)	2 (5.9)
Years of education	7 (3–12)	7 (0–10)	12 (5–14)	7.5 (0–12)	8 (0–14)
Number of pregnancies	4 (1–6)	3.5 (2–6)	3 (1–6)	3 (0–4)	3 (0–6)

**Table 2:** Characteristics of expert group panel participants

Characteristic N (%), median (range)	Expert panel I (10.24.19) *n*=6	Expert panel II (1.23.20) *n*=8	Overall *n*=14
Age in years	59 (40–64)	50 (28–64)	52 (28–64)
Country of birth			
*Kenya*	6 (100)	8 (100)	14 (100)
Religion			
*Protestant*	5 (83.3)	5 (62.5)	10 (71.4)
*Catholic*	0 (0)	1 (12.5)	1 (7.1)
*Moslem*	1 (16.7)	2 (25)	3 (21.4)
Marital status			
*Never married*	2 (33.3)	4 (50)	6 (42.9)
*Currently married*	2 (33.3)	3 (37.5)	5 (35.7)
*Widowed*	2 (33.3)	1 (12.5)	3 (21.4)
*Divorced*	0 (0)	0 (0)	0 (0)
Years of education	16 (15-18)	16 (12-18)	16 (12-18)

### Emergent themes

Below, we present the key themes that emerged in the qualitative work: physical impairment with aging, decreased family support, a turn to religion and social groups, lack of a financial safety net, mixed support from healthcare providers, and stigma as an added burden for women living with HIV.

#### Physical impairment with aging

Participants described several health concerns that they attributed to the aging process. Common physical complains included body pains and arthritis.

*“It is true that as you age the energy that you had reduces. You can’t accomplish the chores that you once did, the speed you had. Now you feel that you are fatigued, your knees are hurting.”* – HIV-negative woman, FGD 3

Hypertension and diabetes were the most common non-communicable diseases reported. Cognitive complaints included memory loss.

*“I can keep keys in a place then begin to look for them everywhere and I can’t find them yet am all alone, and everyone has gone to school … only to find them when I am looking for something else. I told myself age is catching up with me”* – HIV-positive woman, FGD 4

There was such general sharing of physical complaints and health concerns that one woman asked:

*“Is old age sickness*?” – HIV-positive woman, FGD 2

#### Decreased family support

Participants reported changes in their household make-up as they aged, which led to isolation. Many had had spouses who had died or marriages that had dissolved. One woman stated:

“*Apart from not having husbands, some of us don’t even have other people whom we live with.*” – HIV-positive woman, FGD 1

For many, children had moved away after they grew up and were not always supportive, although some continued to visit.

*“An old lady is supposed to be assisted, but nowadays there is no assistance forthcoming from the children, unlike in the days gone by when women used to be assisted.”* – HIV-positive woman, FGD 4

*“For some women their children have rented for them houses and they live apart, but they come around to pay them visits”* – HIV-negative woman, FGD 3

In contrast, women who still had children or grandchildren at home found this to be an important source of motivation and satisfaction:

*“I take care of my child. If it were not for that I would just die”* – HIV-positive woman, FGD 2

#### A turn to religion and social groups

Religious and social groups served as sources of support for participants. Many women reported becoming more religious and participating in church groups.

*“When one gets old that is when one gets too involved in church matters simply because most of her tasks are gone so it’s her and the church.”* – HIV-positive woman, FGD 4

“*There are meetings organized on Tuesdays, Wednesdays, there are some organized by pastors, there are those organized by women to talk about issues of life, so you can get to learn something you didn’t know. You get to learn a lot.*” – HIV-negative woman, FGD 3

“Merry-go-round” groups in which women regularly contributed a small sum to a fund that was paid out to members on a rotating basis were common and helped meet financial needs. While these social support groups were helpful to most women, some women described gossip and back-biting that led them to not participate.

*“There are no groups because you go and get filled with useless talk until when you get out you don’t even understand yourself.”* – HIV-positive woman, FGD 2

#### Lack of a financial safety net

Most participants felt that it was not easy for older women to get help from others when needed. Many said that women must support themselves through work and save money for the future. Government support was minimal and difficult to access:

*“I hear if you go there, they ask for your ID card and if they look at ID, they say you do not qualify.”* – HIV-positive woman, FGD 4

The financial challenges some women experienced led to them not being able to afford more than one meal a day. This led to stress and worry:

*“You can only sleep if you know that you have someone who can provide for you. But for us, if you are aging and you don’t have someone who will provide for you, you start losing sleep.”* – HIV-negative woman, FGD 3

#### Mixed support from healthcare providers

For many women, healthcare providers and clinics were described as the greatest source of support:

“*There are clinics such as this one where they offer health talks; those too work as you educate them in a language they understand”* –HIV-negative woman, FGD 3

Most women found providers to be sources of motivation and support as they navigated their chronic health problems. While interacting with the health system was mostly positive, however, some women complained of not being taken seriously:

*“You see like me, I used to be very sick quite often, and I saw some arrogance. It’s like I had gone there so many times and they were tired of me. Such that when you go there … the doctor says you are not sick … You see, you are ignored...you are ignored and your body is very weak, you are sick.”* – HIV-positive woman, FGD 1

For others, the utility of seeking care was limited by their financial ability to pay for visit fees and medications:

*“For that you need to see the doctor and if you don’t have the money to see the doctor so that you can get help … . and on that I still haven’t sought help.”* – HIV-negative woman, FGD 3

#### HIV stigma as an added burden

Some distinct challenges were reported by women living with HIV. Stigma was common in the community and most women preferred to keep their HIV status a secret. Women feared that if their HIV status became known, people would talk about them, and they would become isolated.

“*Someone will know that truly I have been infected. She will go and tell other people all over in the village.*” – HIV-positive woman, FGD 2

Notably, no women mentioned physical impairments caused by HIV itself, and some women said the challenges that women with and without HIV face as they age are similar.

*“They don’t have HIV but struggling with all the other diseases, so we are all the same, I don’t see a difference.”* – HIV-positive women, FGD 4

##### SVI adaptation

Table 3 outlines the 19 items in the SVI as used by Armstrong and colleagues, our interim SVI expanded to 34 items in order to include additional constructs relevant to the Kenyan context, and the final 16-item SVI-Kenya, with less important items removed after the rating and ranking exercise [13]. The table is organized by domains that reflect the major themes from our qualitative work, with the addition of three domains: safety, instrumental support, and emotional support. These areas grew out of questions in the original SVI and their discussion by the expert panel, rather than from the FGD.

**Table 3:** Components of original SVI, SVI-Kenya draft, and SVI-Kenya final version with scores from ranking process

Domain	Original SVI by Andrew, *et al*.	SVI-Kenya draft from October 2019	SVI-Kenya final	Average importance rating (Range 1-5)	Ranking within domain
Barriers: Physical impairment	Does your hearing cause difficulty when you visit with friends?	Does your hearing cause difficulty when you visit with friends?		1.67	5.17
		Are you able to read?		1.83	4.67
	Are you able to see well enough to recognize a friend across a street?	Are you able to see well enough to recognize a friend across a street? ^b^		2.17	--
Barriers: Physical safety		Have you experienced any physical violence at home?	Have you experienced any physical violence at home?	1.17	2.00
		Do you feel safe at home?	Do you feel safe at home?	1.83	3.00
		Do you feel you have control over things that happen to you?	Do you feel you have control over things that happen to you?	1.5	4.00
		Do you feel safe in your neighborhood? ^b^		2.17	--
	Do you think most people can be trusted?	Do you think most people can be trusted? ^b^		2.83	--
Support from family	Do you live alone?	Do you live alone?	Do you live alone?	1.5	1.00
	How many relatives do you see at least once a month?	How many relatives do you see at least once a month?	How many relatives do you see at least once a month?	1.83	2.00
	What is your marital status? ^a^			--	--
		Do you live in an old people’s home? ^b^		2.67	2
Group participation	Do you participate in any groups?	Do you participate in any social groups, such as “merry-go-rounds”?	Do you participate in any social groups, such as “merry-go-rounds”?	1.67	1.50
		How often do you attend religious services?	How often do you attend religious services?	1.83	2.50
	Do you do any regular work, whether volunteer or paid?^a^			--	--
Instrumental support		If you were sick, is there someone who could take you to see a doctor?	If you were sick, is there someone who could take you to see a doctor?	1.5	1.50
	Do you have anyone to help with things like shopping or picking up medications?	Do you have anyone you can send for shopping or picking up medicine when you need it?	Do you have anyone you can send for shopping or picking up medicine when you need it?	1.83	2.67
	Are you able to find somebody to help with daily chores when needed?	If there is something you need help with at home, are you able to get help? ^b^		2.7	--
	Could you find someone to care for your house or belongings if needed?	In an emergency or if you are not around, can you have someone take care of your belongings? ^b^		2.17	--
Emotional support		Do you feel loved?	Do you feel loved?	1.83	2.17
	Do you have somebody to talk to about important decisions?	Are there people (relatives, neighbors, friends) who you can talk to about important decisions?	Are there people you can talk to about important decisions?	1.67	3.17
	Do you know somebody you can turn to with a personal problem?	Do you know somebody you can turn to with a personal problem?	Do you know somebody you can turn to with a personal problem?	1.33	3.67
	When you are lonely, do you have someone to talk to?	When you are lonely, do you have someone to talk to?	When you are lonely, do you have someone to talk to?	1	4.67
	How many people do you feel close to?	How many people do you feel close to?		1.67	5.17
	How often do you meet or talk with family or friends?	How often do you meet with or talk to family or friends?		1.67	5.67
	Is there at least one person whose advice you trust?	Is there at least one person whose advice you listen to and respect?		1.83	6.33
	Do other people talk to you about decisions important to them?	Are there people (relatives, neighbors, friends) who come to you for advice? ^b^		2.83	--
	How many close friends do you have? ^a^			1.67	--
Financial security		Do you feel that your income is enough to do all your expenses and save?	Do you feel your income is enough to do all your expenses and save?	1.17	3.17
		Are you medically covered?	Are you medically covered?	1	3.83
		Do you receive a pension or other regular income?		1.17	4.30
		In the last 12 months, how often did you run out of money for your basic needs?	In the last 12 months, how often did you run out of money for your basic needs?	1.67	4.50
		In the last 12 months, how often have you had to borrow money from a friend or relative to survive financially?		1.83	5.33
	Approximately how much income does your household have each month?	Approximately how much income does your household have each month?		1	5.67
		Do you receive any services from the government?		1.67	5.67
		In the past four weeks, did you go to sleep at night hungry because there was not enough food?		1.33	6.17
		Would you say that financially your future is secure or uncertain?		1.5	6.30

^a^Removed from the SVI by the expert panel at the initial meeting

^b^Dropped due to an average importance rating of 2 or higher

During the first expert panel meeting (step three), three variables were removed from the SVI draft by the expert panel: marital status, working or volunteering, and having close friends. Regarding marital status, the panel felt this should be collected as a sociodemographic variable but did not think it was informative for evaluating social frailty, given how frequently married couples in Kenya live in separate places for economic reasons. Working in formal employment or as a volunteer was considered very unusual for older women in Kenya, who rarely work in the formal economy and are too poor to volunteer. The item on having close friends was felt to be redundant with the question on how many people one felt close to, so was removed from consideration.

In terms of added variables, a question on ability to read was added to the physical impairment domain, to capture both literacy and vision deficits. In the safety domain, the SVI item on whether most people can be trusted led to a discussion of how physical safety was more important and a basic need. In the family support domain, living in a home for older persons was added for discussion; while such institutions exist in Kenya, the panel considered this a living situation of last resort and said it was rare. In the group participation domain, “merry-go-round” groups were specified and a question on attending religious services added. Regarding the instrumental and emotional support questions, the panel added having someone take you to the doctor and feeling loved, respectively. Finally, given the very low income of most older women in Kenya, several items on financial security were added.

In the final expert panel (step five), ratings and rankings were collected in a spreadsheet, with the physical impairment and safety domains grouped together as “barriers” in a single domain. One panel member’s data were excluded because she clearly did not understand the exercise despite assistance. After average importance ratings were calculated, 7 items rated at 2 or higher were removed from consideration. Once this was done, the items with the highest rankings within a given domain were retained. In only one case was an exception made in determining the final items: the question “In the last 12 months, how often did you run out of money for your basic needs?” was retained instead of the question “Do you receive a pension or other regular income?” despite a higher ranking, in order to capture financial hardship despite a pension or other regular income, which was relatively unusual. Table 4 contains the final SVI-Kenya items, responses, and scoring instructions.

**Table 4:** SVI-Kenya final version

Question	Answer	Score
1. **Do you feel safe at home?**	Yes (0) No (1)	
2. **Do you feel you have control over things that happen to you?**	Yes (0) No (1)	
3. **Have you experienced any physical violence at home?**	No (0) Yes (1)	
4. **Are you medically covered?**	Yes (0) No (1)	
5. **Do you feel your income is enough to do all your expenses and save?**	Yes (0) No (0.5) No income (1)	
6. **In the last 12 months, how often did you run out of money for your basic needs?**	Never (0) Once or twice (0.33) Few (0.67) Many (1)	
7. **Do you have anyone you can send for shopping or picking up medicine when you need it?**	Yes (0) No (1)	
8. **If you were sick, is there someone who could take you to see a doctor?**	Yes (0) No (1)	
9. **Do you live alone?**	No (0) Yes (1)	
10. **Do you participate in any social groups, such as "merry-go-rounds"?**	Yes (0) No (1)	
11. **How many relatives do you see at least once a month?**	Many (0) Few (0.5) None (1)	
12. **How often do you attend religious services?**	Daily (0) Sometimes (0.33) Weekly (0.67) Never (1)	
13. **Do you feel loved?**	Yes (0) No (1)	
14. **Are there people (relatives, neighbours, friends) who you can talk to about important decisions?**	Yes (0) No (1)	
15. **Do you have someone you can go to with a personal problem?**	Yes (0) No (1)	
16. **When you are lonely, do you have someone to talk to?**	Yes (0) No (1)	

**Scoring:** Each item assessed in the SVI-Kenya is expressed as the presence or absence of a deficit, with scores ranging from 0 (no deficit) to 1 (maximal deficit). Scores for each item should be summed and divided by the number of questions to create an overall score ranging from 0% to 100%.

## Discussion

Due to the lack of a standardized, validated tool to measure social frailty in older adults in East Africa, we sought to adapt an existing measure to the Kenyan context. In reviewing the literature on social vulnerability, we identified the theoretical framework described by Bunt and colleagues as likely to be relevant [10]. Bunt and colleagues used the theory of Social Production Function to conceptualize social frailty in a model with four domains: social needs fulfilment, social resources, general resources, and social behaviors or activities [10, 17]. Of the numerous published indices Bunt and colleagues identified and reviewed, only the SVI developed by Andrew and colleagues captured all four domains [18]. We therefore selected the SVI as our measure to adapt.

The process we used to adapt the SVI included qualitative work exploring the lived experiences of older Kenyan women. The themes we identified suggest that as women age and develop health conditions that limit their physical abilities, they are often also struggling with decreased family support and a lack of financial security. Those who have supportive families or support from religious and social groups fared better. This finding is in keeping with a study of older adults living in Nairobi slums, in which women were found to have more and stronger social ties than men, which put them at an advantage as physical impairments eventually reduced women’s ability to do chores [9]. As in our study, older adults living with HIV in Western Kenya reported challenges navigating the healthcare system and finding supportive providers [19]. While recent studies of older adults in Kenya have focused those living with HIV, our study adds to the literature by incorporating the experiences of HIV-negative women, broadening perspectives to include challenges to older women regardless of HIV status.

Working with the panel of experts, we expanded the original 19-item SVI to include additional items the experts thought important and drop items less relevant in Kenya. While the final domains for the SVI-Kenya are similar to those of the original SVI, hearing and vision were deemed less important barriers than concerns about physical safety, and so physical safety rather than impairment is included in the SVI-Kenya. Of note, gender-based violence is common in Kenya and has reportedly increased during the COVID-19 pandemic [20]. Similar concerns exist about elder abuse in Africa and have led to calls for more research in this area [21]. Other adaptations included questions focused on participation in religious and social groups that help women save money (i.e., “merry-go-rounds”). While SVI items on instrumental and emotional support were retained with no changes, the domain that led to the greatest expansion of items was financial security. This focus is in keeping with data on the high rates of poverty and food insecurity among older women in Africa [7, 8]. In order to make the SVI status-neutral, we did not include questions related to HIV stigma, for which existing scales are available [22]. In addition, support from healthcare providers was not incorporated into the SVI specifically, but access to affordable, high-quality care is an important need that should be addressed in future work.

We are currently using the SVI-Kenya in a cross-sectional study in an urban setting comparing 150 women over age 40 living with HIV to 150 women living without HIV. The study’s objectives are to characterize social frailty using the SVI-Kenya and a social network survey, to evaluate associations between SVI scores and both clinical frailty measured with the Rockwood 7-point Clinical Frailty Scale and disability measured with the World Health Organization Disability Assessment Schedule (WHODAS)-12, version 2 [23, 24]. We will evaluate whether HIV status modifies the association between SVI and either clinical frailty or disability. Finally, we will explore the association between SVI score category and HIV treatment adherence and viral suppression among the 150 participants living with HIV. This work will provide valuable data on the social vulnerability of older women in Kenya and form the basis for future work to develop interventions that could improve outcomes.

The strength of the current work is its grounding in Social Production Function theory and use of the SVI, which has demonstrated associations with increasing age, female sex, and adverse outcomes including clinical frailty, cognitive decline, and mortality in other settings [10, 12, 13, 17, 18, 25, 26]. We followed an approach used previously to adapt the SVI for the Dutch context [15] but strengthened this approach through the use of FGDs involving 34 older women, many of them living with HIV. Our study also has limitations. While our initial work focused on women aged 50 and older, we broadened inclusion criteria to those aged 40 and above due to a focus on evaluating social frailty over a range of ages in our ongoing work and challenges recruiting women at older ages. In addition, while the SVI-Kenya has face validity, it needs further validation before use in other studies. Its generalizability to other settings and populations, including older Kenyan men, is not known.

Overall, the SVI-Kenya promises to be a useful instrument for the assessment of social frailty among older women in Mombasa, Kenya. To our knowledge, it is the only measure of social frailty that has been adapted specifically for use in sub-Saharan Africa, the region most impacted by the HIV pandemic and one in which older adults, especially women, could use additional support. If the SVI proves to perform well in practice and to be correlated with other social support and frailty measures in our ongoing study in Kenya, it could be used more widely, and adapted to specific settings as needed.

## Data Availability

Deidentified data will be made available in the Harvard Dataverse (https://dataverse.harvard.edu/) upon publication. The qualitative data used and/or analysed during the current study are available from the corresponding author on reasonable request.
